# Climate change – a burning topic for public health

**DOI:** 10.25646/10387

**Published:** 2022-08-31

**Authors:** Lothar H. Wieler

**Affiliations:** Robert Koch Institute, Berlin President

The climate crisis is arguably the greatest threat to human health and well-being. As illustrated by the COVID-19 pandemic, rapid, but sustainable, action is crucial in public health crises and in the climate crisis no individual or health system will be left unaffected. While the world’s attention was focussed on the ongoing acute COVID-19 crisis, the health effects of human-induced climate change continue to accumulate [1]. Public health has many opportunities to proactively address the health impacts of climate change and public health experts play a crucial role in the prevention of climate-related diseases [2]. Consequently, as the national public health institute, the Robert Koch Institute (RKI) has an important role to play in climate change adaptation and mitigation.

The RKI has longstanding expertise in the field of One Health which has been guiding the institute’s work on zoonotic diseases and antimicrobial resistance as well as wider areas such as environmental health for a number of years. To further strengthen its existing expertise and build new capacities, the RKI has recently extended its focus and is increasing its efforts in the area of effects of climate change on health. In 2022, the RKI established its Office for Climate Change and Health, responsible for coordinating all internal and external activities as well as external requests and cooperation regarding this subject. Under the institute’s cross-departmental working group on climate change and health, existing activities will be drawn together, thereby promoting internal exchange, cooperation and cohesion. The goal of the RKI is to strategically intensify its work in the area of climate change and health and to become a ‘key climate actor’ [3].

There is still much that needs to be done and it cannot be done alone. To address this complex issue, diverse cross-sectoral knowledge and networks are needed, in order to exchange knowledge and approaches. Therefore, the RKI closely collaborates with other national public health institutes through the International Association of National Public Health Institutes’ (IANPHI) Committee on Climate Change and Health. To further signal the institute’s perception of climate change as a major health threat, for this year’s Robert Koch Colloquium (RKC), the RKI put the spotlight on ‘Climate change and public health’ to share the knowledge of renowned experts in this field. This edition of the Journal of Health Monitoring focusses on the RKC 2022 which addressed the effects of climate change on population health and potential adaptation and mitigation solutions. The Colloquium was composed of five lectures and concluded with a panel discussion. Each lecture touched upon a specific aspect of the relationship between climate change and health.

Sabine Gabrysch from the Potsdam Institute for Climate Impact Research (PIK) spoke about how human health fundamentally depends on the functioning of ecosystems and a stable climate, by diagnosing the planetary health crisis. She concluded by proposing a positive transformation towards healing, well-being and quality of life including the people and the planet (see Gabrysch 2022). Arturo Casadevall of the Johns Hopkins School of Public Health, addressed the connections between microbial virulence, mammals and climate change. The speaker presented the hypothesis that fungal diseases contributed to both the extinctions at the end of the Cretaceous geological epoch that resulted in the demise of the dinosaurs and to the great mammalian radiation that followed in the tertiary era. This lecture touched on possible consequences of climate change for the emergence of new fungal diseases as fungal species adapt to a warmer world (see Casadevall 2022). Lyle Petersen from the Centers for Disease Control and Prevention (CDC) spoke about climate change and vector-borne diseases, using the examples of mosquito- and tickborne diseases. His lecture explored current concepts of how climate change is expected to impact the emergence of vector-borne disease in the context of the many other interacting biological, ecological, and sociological drivers of such diseases (see Petersen et al. 2022). Aleksandra Kazmierczak from the European Environment Agency (EEA) spoke about climate-related hazards and their implications for health and well-being, especially focussing on vulnerable groups in Europe (see Kazmierczak 2022). Lastly, Maria Neira from the World Health Organization (WHO) presented the health argument for climate action, stressing that climate mitigation and adaptation is public health prevention. She reiterated the importance of health co-benefits in climate change mitigation, the importance of health (equity) in all policies and ended with ten prescriptions for action. The RKC 2022 concluded with a panel discussion which brought together Daniela Jacob (Director of the Climate Service Center Germany), Dirk Messner (President of the German Environment Agency), Thomas Mettenleiter (President of the Federal Research Institute for Animal Health) and Lothar H. Wieler (President of the Robert Koch Institute), who discussed possible next steps for climate change and health in Germany. Moderated by Maike Voss (Managing Director of the Centre for Planetary Health Policy), the key aspects addressed included climate change-related challenges facing the public health community in Germany, concrete tasks of the German public health community to protect the climate and mitigate the effects of climate change on health, the importance of cross-institutional and sectoral collaboration, the contribution of the RKI and the recommendations for action for public health from a scientific perspective as well as next steps.

The RKI can be a key actor in the field of climate change and public health through its evidence-based scientific recommendations on topics such as heat mortality, nutrition and physical activity and health behaviour but also through its surveillance and monitoring activities. Health monitoring and surveillance will play a major role in identifying epidemiological developments in time as well as in understanding causes and correlations. Risk analyses, health reporting, communication and collaboration must accompany all of these activities. Finally, it is important to set an example by reducing the institute’s CO_2_ footprint with the RKI’s concept of ‘RKI Greening’.

As the natural historian and biologist, David Attenborough, once said ‘Self-interest is for the past, common interest is for the future’. To succeed in the task ahead, this has to become the guiding principle.
